# About “Efficacy of bispectral index monitoring for prevention of anesthetic awareness and complications during oocyte pick-up procedure”

**DOI:** 10.3906/sag-1711-201

**Published:** 2019-02-11

**Authors:** Ayşe Çiğdem TÜTÜNCÜ

**Affiliations:** 1 Department of Anesthesiology and Reanimation, Faculty of Medicine, Cerrahpaşa University, İstanbul Turkey

**To the Editor,**

I read the article “Efficacy of bispectral index monitoring for prevention of anesthetic awareness and complications during oocyte pick-up procedure” written by Urfalıoğlu et al. (1) in the *Turkish Journal of Medical Sciences* with great interest. The authors have applied sedation with propofol infusion and sevoflurane during the oocyte pick-up (OPU) procedure and compared the efficacy of bispectral index (BIS) monitoring in preventing awareness during anesthesia. BIS level, amount of hypnotic consumption, and recovery period (beginning when patients woke after the procedure until the modified Aldrete score was ≥8 in the recovery unit) have been used as parameters of the study. The scoring system used during the recovery period is the Aldrete scoring system which is the most commonly used scoring system in the recovery units during the postoperative period after anesthesia procedures and evaluates respiration, circulation, and oxygenation besides the consciousness of the patient. If the primary objective of the study is considered to compare awareness possibility during the procedure, and the amount of hypnotic administered by using BIS monitoring or in the control group during propofol and sevoflurane sedation, we think that the use of scoring systems, such as Ramsay Sedation Scale, the sedation Visual Analog Scale, or the Observer’s Assessment of Alertness/Sedation Scale, that are recommended to be used in similar procedures, instead of the recovery scoring system, will be more suitable for the purpose of the study and will allow to perform goal-directed evaluations both during the procedure and in the recovery period (2,3).

Moreover, it was indicated that 10–20 mg of propofol intravenous (IV) bolus was additionally used in the control group in the cases of conventional reactional responses after using the induction dose of propofol (2 mg/kg) in both groups. It was not indicated how this dose was determined (such as weight, body surface area), and again a BIS value of 60 and above was accepted as reference in the BIS group and the same dose has been administered; we think that indicating the additional dose of propofol given in table 1 in the form of drug dose/determined parameter is important as an indicator in terms of the hipnotic consumption.

Furthermore, the article did not indicate how many patients required an additional propofol dose and the number of patients who required multiple (repetitive) additional doses. We also think that comparing the number of patients who required additional hypnotic will be an important indicator in determining the efficacy of BIS.

We also would like to add that monitoring the end-tidal anesthetic gas concentrations in the control and BIS groups, and the comparison of these values for both groups, especially the changes in the end tidal gas concentration in patients requiring additional dose, will provide important information to the anesthesiologist in monitoring the sedation depth and awareness during the procedure (4).
